# Editorial: The relationship between sarcopenia and metabolic diseases: Its formation mechanism and intervention means

**DOI:** 10.3389/fendo.2022.972238

**Published:** 2022-08-25

**Authors:** Qingfeng Cheng, Chaodong Wu, Lixin Guo, Jinbo Hu

**Affiliations:** ^1^ Department of Endocrinology, The First Affiliated Hospital of Chongqing Medical University, Chongqing, China; ^2^ Department of Nutrition, Texas A&M University, College Station, TX, United States; ^3^ Department of Endocrinology, Beijing Hospital, National Center of Gerontology, Institute of Geriatric Medicine, Chinese Academy of Medical Sciences, Beijing, China

**Keywords:** sarcopenia, intervention, aging, mechanism, metabolic diseases

Sarcopenia is an age-related skeletal muscle wasting syndrome (1), derived from the Greek words sarx (muscle) and penia (loss). Muscle loss starts at the age of 30, and, an individual, if he or she doesn’t exercise, maybe lose 10 percent of his or her muscle mass by age 50. On an average, muscle mass decreases by about 5 kg every 10 years after the age of 40 (2). The term sarcopenia was first proposed by Dr. Irwin Rosenberg of Tufts University in 1989 (1). Delmonico et al. first used Dual energy X-ray Absorptiometry (DXA) to measure muscle mass in 1998 (3). It is suggested that the muscle mass of young individuals is lower than 2 standard deviations for sarcopenia.

The main clinical manifestations of sarcopenia are progressive, reduced muscle mass, and/or decreased muscle strength or decreased muscle physiological function. Sarcopenia is closely associated with metabolic and cardiovascular diseases, with increased risk of falls, fractures, disability, hospitalization and mortality in older persons, and can increase the medical, social and economic burden (4–7). Also, sarcopenia is a complex disease involving environmental and genetic factors, and its occurrence involves many risk factors and mechanisms. Current studies indicate that sarcopenia caused by aging is mainly related to nutrition, exercise, related hormones, inflammation, cytokines, mitochondrial abnormalities, muscle satellite cells and autophagy, as well as miRNA, genetics and other factors (8–11). In addition, sex may also be a factor in sarcopenia (Merchant et al.).

After the emergence of the word sarcopenia, numerous research results have been produced, and various associations have been proposed as diagnostic criteria for sarcopenia (12–14). However, there is a lack of the universally recognized indicators as biological markers of sarcopenia. Some researchers have proposed that irisin can be used as a biological marker of sarcopenia (15). Dai et al explored potential biomarkers of sarcopenia from the perspective of amino acid spectrum. The dynamic balance of Amino Acid Flux in Sarcopenia is critical for maintaining muscle health (16).

Treatment of sarcopenia is mainly non-pharmacological. For instance, resistance exercise and nutritional supplements are the most widely accepted strategies. A growing number of studies combine exercise with nutrient (protein and amino acids) intake, and suggest that exercise alone is more effective at interfering with sarcopenia (17). Nutritional interventions include adequate intake of protein, vitamin D, antioxidant nutrients and long-chain polyunsaturated fatty acids. Protein is essential for improving muscle mass and strength, and one study suggested a significant correlation between leg muscle and strength and a frequent intake of protein (30 g or more per meal, 2 times a day) (18). Drugs currently being developed in clinical trials may reverse age-related loss of muscle mass and function. Regarding androgens/androgen receptor modulators, it is suggested that androgens increase muscle mass and muscle protein synthesis and decrease ubiquitin-ligase expression (19, 20). Guligowska et al. found that sarcopenia was closely related to gonadotropin, sex hormone and DHEAS levels in elderly men. If the study is confirmed in a larger population, the results obtained may shift the paradigm of drug intervention strategy of sarcopenia (21). The Ghrelin secret-promoting agent capromorelin increases weight and muscle mass in older adults and improves walking and stair climbing (22, 23). These data suggest that Ghrelin has a potential therapeutic effect on sarcopenia, but more prospective studies are needed to confirm it. Other drugs, such as metformin, anti-Myostain Activin ii receptor, estrogen, and angiotensin converting enzyme inhibitors, are all being studied for intervention of sarcopenia (20). Other potential targets include PGC-1A, phosphodiesterase inhibitors (PDE), skeletal muscle stem cells, and gene recombination (20, 22, 23).

In summary, the research on sarcopenia is still in its exploratory stage, and the pathogenesis of sarcopenia is not well understood. For future studies, it is necessary to enhance the efforts on elucidating the underlying cellular and molecular mechanisms, developing validate biomarkers, improving the accuracy of diagnostic tests, solving the problem of consistency of diagnostic criteria, and designing effective strategies for the prevention and treatment of sarcopenia. The editors thank all authors and reviewers for their scientific contributions.

## Author contributions

Written by QC, revised by JH, CW, and LG. All authors contributed to the article and approved the submitted version.

## Acknowledgments

The editors thank all authors and reviewers for their scientific contributions.

## Conflict of interest

The authors declare that the research was conducted in the absence of any commercial or financial relationships that could be construed as a potential conflict of interest.

## Publisher’s note

All claims expressed in this article are solely those of the authors and do not necessarily represent those of their affiliated organizations, or those of the publisher, the editors and the reviewers. Any product that may be evaluated in this article, or claim that may be made by its manufacturer, is not guaranteed or endorsed by the publisher.
